# Human Bronchial Epithelial Cells Induce CD141/CD123/DC-SIGN/*FLT3* Monocytes That Promote Allogeneic Th17 Differentiation

**DOI:** 10.3389/fimmu.2017.00447

**Published:** 2017-04-25

**Authors:** Amiq Gazdhar, Fabian Blank, Valerie Cesson, Alban Lovis, John David Aubert, Romain Lazor, Francois Spertini, Anne Wilson, Katrin Hostettler, Laurent P. Nicod, Carolina Obregon

**Affiliations:** ^1^Department of Pulmonary Medicine, University Hospital Bern, Bern, Switzerland; ^2^Department of Clinical Research, University of Bern, Bern, Switzerland; ^3^Pneumology Division, Centre Hospitalier Universitaire Vaudois, Lausanne, Switzerland; ^4^Immunology and Allergy Division, Centre Hospitalier Universitaire Vaudois, Lausanne, Switzerland; ^5^Department of Fundamental Oncology, University of Lausanne, Epalinges, Switzerland; ^6^Clinics of Respiratory Medicine, Department of Biomedicine, University Hospital of Basel, Basel, Switzerland

**Keywords:** bronchial epithelial cell-conditioned media, monocytes, monocyte-derived dendritic cell, Th17, interleukin-17, IL-1β, *FLT3*

## Abstract

Little is known about monocyte differentiation in the lung mucosal environment and about how the epithelium shapes monocyte function. We studied the role of the soluble component of bronchial epithelial cells (BECs) obtained under basal culture conditions in innate and adaptive monocyte responses. Monocytes cultured in bronchial epithelial cell-conditioned media (BEC-CM) specifically upregulate CD141, CD123, and DC-SIGN surface levels and *FLT3* expression, as well as the release of IL-1β, IL-6, and IL-10. BEC-conditioned monocytes stimulate naive T cells to produce IL-17 through IL-1β mechanism and also trigger IL-10 production by memory T cells. Furthermore, monocytes cultured in an inflammatory environment induced by the cytokines IL-6, IL-8, IL-1β, IL-15, TNF-α, and GM-CSF also upregulate CD123 and DC-SIGN expression. However, only inflammatory cytokines in the epithelial environment boost the expression of CD141. Interestingly, we identified a CD141/CD123/DC-SIGN triple positive population in the bronchoalveolar lavage fluid (BALF) from patients with different inflammatory conditions, demonstrating that this monocyte population exists *in vivo*. The frequency of this monocyte population was significantly increased in patients with sarcoidosis, suggesting a role in inflammatory mechanisms. Overall, these data highlight the specific role that the epithelium plays in shaping monocyte responses. Therefore, the unraveling of these mechanisms contributes to the understanding of the function that the epithelium may play *in vivo*.

## Introduction

Over the last few years, there is compelling evidence that the epithelium not only plays a role as a barrier but also that epithelial cells (ECs) and their microenvironment are also capable of modulating immune functions (1–3). An interesting aspect is the observation that the environment of lung or intestinal ECs generate dendritic cells (DCs) with regulatory features (4–6). However, little is known about the role of lung ECs soluble components in monocyte function, and essential questions remain open with regard to monocyte/DC differentiation (7, 8).

Monocytes are phagocytes with an important innate immune response toward pathogens (9). This population is heterogeneous and has an important plasticity, which makes it capable of giving rise to different well-defined populations, such as macrophages or DCs *in vitro*. *In vivo*, it has been reported that extravascular monocytes are able to retain their monocytic features with minimal differentiation and appear to support DC function (10). In spite of the fact that it has been difficult to properly identify monocytes and both cell-derivates *in vivo*, in the mouse model, a phenotype of monocyte-derived DCs (ModDCs) has been identified under both the steady and inflammatory states (11–14). Little is known, however, about human ModDCs. Human monocytes can be classified according to their expression of CD14 and CD16 (FcγR-III): classical (CD14^+^CD16^−^), intermediate (CD14^+^CD16^+^), and patrolling (CD14^dim^CD16^+^) monocytes (9, 15). In regard to DCs, three main DC subsets have been described in human blood: two conventional DC subsets, which can be identified in the HLA-DR^+^CD14^−^ fraction by the expression of CD1c (BDCA-1) and CD141^high^ (BDCA-3), shown to originate from a common DC progenitor (16, 17), and the pDC subset which is identified by CD303 (BDCA-2) CD304 (BDCA-4) and CD123^bright^ expression. In contrast to the well-defined phenotypic expression of blood DCs, in human lungs, there is evidence for high phenotypic heterogeneity. In addition to the BDCA populations, three other subpopulations can be distinguished by the expression of langerin, CD1a, and DC-SIGN, but these markers are promiscuously expressed, making it difficult to differentiate specific populations (18). For example, studies show that the Langerin^+^ population in lung tissues can express BDCA-1 and CD1a, but not BDCA-3 (18). In contrast, in the lung tissues of allergic asthma patients, most of the BDCA1^+^cDCs express BDCA-3 (19), suggesting that the DC phenotype can be modulated by the epithelial environment. It was recently demonstrated that gene expression profiling and mass cytometry analysis are useful techniques for the identification of lineage-imprinted cell markers in human tissues, such as CD172α/IRF4 and XCR1/IRF8, thus allowing for a clear discrimination between CD1c^+^CD14^−^ DC and CD141^+^CD14^−^ DC subsets, respectively (20). In regard to ModDCs in the lungs, they have been poorly investigated due lack of specific markers. Recent studies, however, at steady state found CD1c^+^ cells on the HLA-DR^+^CD14^+^ fraction in lung tissues and Bronchoalveolar lavage fluid (BALF). These cells were related to a subtype of ModDCs enriched for the gene signatures of ModDCs described in the literature, which includes the expression of *ZBTB46, IRF4*, and *FLT3* genes and the surface markers CD206 and CD1a (21). A similar phenotype was described in arthritic synovial fluid, which was associated with inflammatory DCs by its capacity to induce Th17 cell differentiation, *ex vivo* (22). However, for lung diseases, particularly in chronic inflammation, ModDCs have not yet been identified.

In the present study, we demonstrate how ECs differentially regulate the phenotype and function of monocytes and as a result stimulate IL-17/IFN-γ-producing naive T cells and IL-10-producing memory T cells. This particular phenotype is characterized by the surface expression of CD141/CD123/DC-SIGN and *FLT3* gene expression. Furthermore, we have characterized a CD141/CD123/DC-SIGN triple-positive population in patients with sarcoidosis, which expresses IRF4, thus demonstrating that this phenotype increases during inflammation and may be related to ModDCs. Our findings contribute to the understanding of the mechanism by which the epithelium modulates monocyte function, relevant to the understanding of epithelial dysfunction during chronic inflammation.

## Materials and Methods

### Monocyte and Epithelial Cell Isolation and Preparation

PBMCs were isolated by Ficoll-Paque density gradient centrifugation. Monocytes were prepared as described previously (23, 24) and were characterized by high expression of CD14 (more than 86%). Differentiation of DCs from monocytes was performed as originally described by culturing cells in the presence of a granulocyte-macrophage colony-stimulating factor (10 ng/mL) and interleukin-4 (10 ng/mL) for 4 days (25). Differentiation of macrophages from monocytes was performed by culturing cells in the presence of 100 ng/mL of macrophage colony-stimulating factor (Prepotech, UK) and 10 mM HEPES (AMIMED, Switzerland) for 7 days.

Bronchial epithelial cells (BECs) were grown from a BEAS-2B cell line (number CRL-9609; ATCC). Cell cultures were maintained in LHC-8 (Gibco, Thermo Fisher, Reinach, Switzerland) supplemented with 2.2 μM epinephrine (Epi) (Sigma-Aldrich, Buchs, Switzerland) and 0.3 nM retinoic acid (RA) (Biofluids, Gaithersburg, MD, USA). Before the experiments, BECs were grown in monolayers to 80–85% confluence in 75-cm^2^ culture flasks. Then, the culture medium was removed, and cells were washed with PBS. BECs were subsequently cultured in LHC-8 without Epi and RA. After 48 h, the bronchial epithelial cell-conditioned media (BEC-CM) was harvested, filtered through a 0.22-μm pore-sized filter, and frozen at −20°C until it was used in the experiments. For long-term storage BEC-CM was frozen at −80°C.

Human primary nasal epithelial cells (PNECs) were obtained by brushing the inferior surface of the middle turbinate of both nostrils using a cytological brush (Dent-o-care, London, UK) as described in supplemental experimental procedures. Briefly, cells were cultured in a bronchial epithelial basal medium (BEBM), (Lonza, Switzerland), until they developed into an air-liquid interface. Afterward, BEBM was removed and replaced by PneumaCult™-ALI (STEMCELL Technologies™). PNEC-CM was obtained after culturing cells in their optimal culture conditions for 48 h. Human primary alveolar epithelial cells (PAECs) were cultured from the tissue of patients undergoing surgical lung resection due to lung cancer. Lung tissue was obtained from sites away from the tumors. After the biopsy, the tissue was cut into small pieces of 1 mm^3^, and these were placed into pre-wetted 25 cm^2^ cell culture flasks (Falcon, Corning Incorporated, USA) for cell sprouting. No additional coating was applied. The epithelial growth medium (CELLnTEC, Bern, Switzerland) was replaced every fourth day. AECs were grown under standard conditions (37°C, 21% O_2_, 5% CO_2_). PAEC-CM was obtained after culturing cells in their optimal culture conditions for 24 h. Subsequently, all harvested condition media were centrifuged at 1,200 rpm for 10 min, filtered through a 0.22-μm pore-sized filter, and frozen at −80°C until they were used in the experiments.

### BALF Samples

Bronchoalveolar lavage fluid (BALF) samples were obtained from patients undergoing bronchoscopy for medical reasons by the CHUV Pneumology Service. In total, 4 BALF samples were obtained from patients with biopsy and/or cytology proven adenocarcinoma, 13 from patients diagnosed with sarcoidosis, and 7 from patients with idiopathic or secondary interstitial pneumonia. All of the diseases were diagnosed based on established standard criteria (26–28). Flexible bronchoscopy was performed according to established guidelines as previously described (29, 30). Lavage was performed in 50 mL aliquots with a total amount of 150–250 mL of 0.9% sterile saline. The BALF was recovered by gentle aspiration. Collected samples (20–30 mL) not used for diagnosis were filtered through a nylon mesh filter and cells were counted manually using trypan blue. Cells were centrifuged and the pellet was washed with cold HBSS. Around 1 × 10^6^ cells were prepared per condition and subsequently stained with Abs or isotype controls for flow cytometric analysis.

### Monocyte and DC Stimulation

Human monocytes or DCs (1 × 10^6^ cells/condition) were cultured for 48 h with medium alone or with the conditioned media (CM) obtained from BECs or primary cells, such as PNECs and PAECs. In some experiments, after 24 h of culture, cells were stimulated with 100 ng/mL of lipopolysaccharide (LPS, DIFCO *E. coli* 055:B5) and/or with 0.5 μg/mL anti-CD141 (Miltenyi Biotec, Germany). Mouse IgG1 isotype control (0.5 μg/mL) (eBioscience, Switzerland) was used as a control. To induce an inflammatory environment, monocytes were cultured for 48 h with medium alone or with BEC-CM with 1 ng/mL of the following cytokines, designated as the inflammatory cocktail: human recombinant IL-6 (Invitrogen, Reinach, Switzerland), IL-8, IL-15, IL-1β, GM-CSF (R&D System, Abingdon, UK), and TNF-α (Roche, Basel Switzerland). For the control, cells were supplemented with 100 pg/mL of IL-6 (Invitrogen, Switzerland) and 500 pg/mL IL-8 (R&D System, UK).

### Cytokine Measurements

IL-17, IL-6, IL-8, IL-1β, IL-12p70, IL-10, and TNF-α cytokines were measured in the Luminex Bio-Plex 200 System (Bio-Rad, USA), using Bio-Rad kits according to the manufacturer’s instructions. IL-23 was measured with a commercial ELISA kit (Biotest, France).

### Mixed Lymphocyte Reaction

Naive and memory CD4 T cells were isolated from healthy PBMCs by negative selection using the Naive CD4^+^ T Cell Isolation Kit II or the Memory CD4^+^ T Cell Isolation Kit (Miltenyi Biotec, Germany), respectively. Treated monocytes (cultured with medium alone or with BEC-CM) were co-cultured at a monocyte:T-cell ratio of 1:10 for 9 days, supplemented with IL-2 for the last 2 days and then stimulated for 5 h with PMA and ionomycin. Supernatants were collected and stored at −80°C until they were needed for cytokine measurement. Neutralizing experiments were performed using the following neutralizing antibodies: IL-1β (CRM56, Ebioscience) used at 2 μg/mL and IL-6R (Tocilizumab-Roche) (humanized IgG1) used at 10 μg/mL. Human IgGκ isotope (Sigma, Switzerland) and mouse IgG1 isotype functional grade purified control (eBioscience, Switzerland) were used as controls.

### Flow Cytometry

Cell suspensions were stained with LIVE/DEAD^®^ Fixable Dead Cell Stain (molecular probes) followed by surface staining with the following Abs: anti-FITC-CD1c, APC-CD141, FITC-CD123, PE-DC-SIGN (Miltenyi Biotec), PercPcy5.5-CD14, A700-CD11b, PE-CD11c, FITC-CD80, PB-CD3 (BD Bioscience) KO-CD16, Pe/Cy7-HLA-DR (Beckman Coulter), PE-CD163, PB-CD86, PB-CD56, PB-CD19 (Biolegend), CCR6 (R&D), or isotype-matched control antibodies. In brief, cells were stained for 20 min at 4°C in FACS buffer and fixed in 1% paraformaldehyde. For intracellular staining, cells were fixed and permeabilized with cytofix/cytoperm (BD Bioscience) for 30 min, washed, and labeled by PE-IRF4 (Biolegend) in Perm/Wash buffer (BD Bioscience). Cell-surface fluorescence intensity was assessed on a Gallios flow cytometer (Beckman Coulter) and analyzed using FlowJo (TreeStar). The Mean Fluorescence Intensity ratio was defined as the ratio of specific markers to the corresponding isotype controls.

### Real-time Quantitative RT-PCR

Total RNA was extracted with the RNeasy mini kit (Qiagen Inc.) according to the manufacturer’s instructions. cDNA was synthesized using an iScript™ cDNA Synthesis Kit (Biorad, USA). Transcripts were quantified by the real-time quantitative PCR CFX96 Touch Real-Time PCR Detection System (Bio-Rad) using the master mix iTaq™ Universal SYBR^®^ Green Supermix (Bio-Rad). After amplification, Cq values were obtained using the CFX Manager™ software 2.1 (Bio-Rad). Relative human gene expression of ZBTB46 (qHsaCED0044323), CSF1R (qHsaCID0010604), FCER1A (qHsaCID0005954), IRF4 (qHsaCED0004445), *FLT3* (qHsaCID0021972), XCR1 (qHsaCED0002997), and IL1B (qHsaCID0022272) was assessed by normalizing gene expression values to those of the reference gene β-2-microglobulin (B2M) (qHsaCID0015347).

### Statistical Analysis

Results are expressed as means ± SEM. Multiple group comparisons were made by the analysis of variance test. Each analysis was followed by multiple comparisons test as mentioned in each figure. Comparisons between two groups were made using the two-tailed Student’s *t*-test. The statistical significance was determined using the Mann–Whitney test. *P*-values less than 0.05 were considered statistically significant. All statistical analyses were done with the GraphPad Prism software (GraphPad Software, Inc., La Jolla, CA, USA).

## Results

### BEC-CM Induced a Particular Monocyte Phenotype, Which Is Characterized by the Expression of CD141, CD123, and DC-SIGN

**Figure 1:**
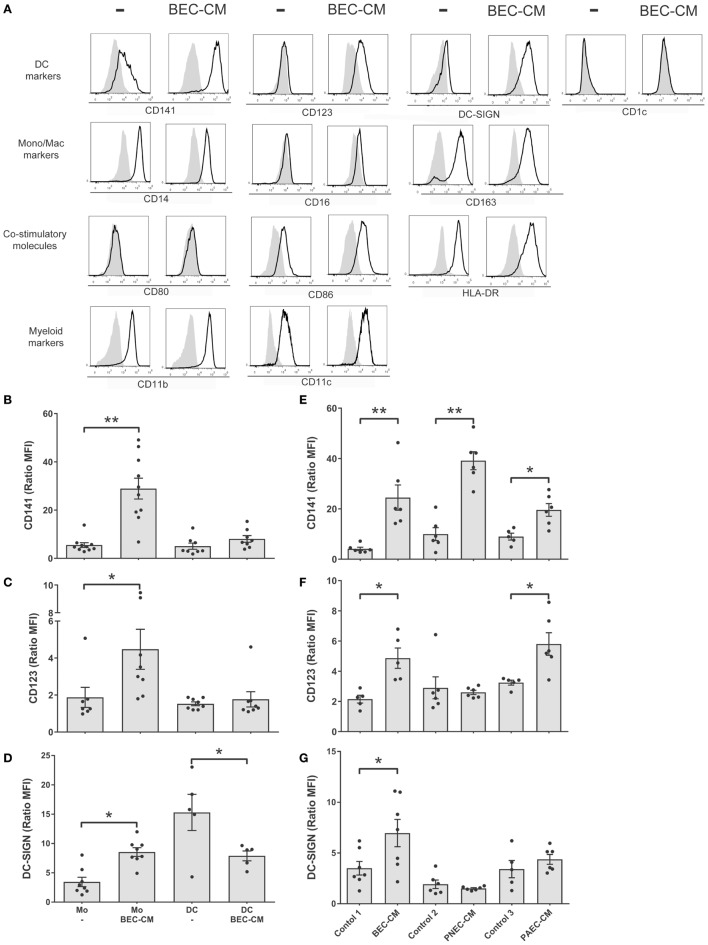
**Bronchial epithelial cell-conditioned media (BEC-CM) enhances the expression of CD141/CD123/DC-SIGN on monocytes**. **(A)** Phenotypic analysis of surface markers on monocytes cultured withBEC-CM. FACS analysis was performed on monocytes that were cultured for 48 h with medium alone or with BEC-CM. Representative histograms (thick lines) of the indicated antibody staining are plotted with the corresponding isotype controls (shaded histograms). All data were obtained with similar results in at least four independent experiments. **(B–D)** Dendritic cells (DCs) were obtained by culturing monocytes with GM-CSF (10 ng/mL) and IL-4 (10 ng/mL). Then, monocytes and DCs were cultured for 48 h with medium alone or with BEC-CM. Bar histograms of the mean fluorescence intensity (MFI) ratio calculated with its specific isotype for **(B)** CD141, **(C)** CD123, and **(D)** DC-SIGN. Data are expressed as means ± SEM of at least eight independent experiments for monocytes and at least five independent experiments for DCs. Statistical analysis was performed using analysis of variance test (ANOVA) (followed by Tukey multiple comparisons test) (*P < 0.05, **P < 0.01). **(E–G)** Monocytes were cultured for 48 h with BEC-CM, PNEC-CM, or PAEC-CM or with the corresponding control media 1:LHC-8, 2: PneumaCult™-ALI, 3: CELLnTEC. Data represent the MFI ratio calculated with its specific isotype for CD141 **(E)**, CD123 **(F)**, and DC-SIGN **(G)**. Data are expressed as means ± SEM of at least five independent experiments. Statistical analysis was performed using ANOVA (followed by Tukey multiple comparisons test), *P < 0.05, **P < 0.01.

In order to analyze the effect of the epithelial soluble component on monocytes, isolated human monocytes were cultured for 48 h in supernatants obtained from human bronchial epithelial BEAS-2B cells (BEC). Flow cytometry was used to analyze the expression of monocyte and macrophage markers, such as CD14, CD16, and CD163; DC markers such as, CD141, CD123, DC-SIGN, and CD1c; co-stimulatory molecules, such as HLA-DR, CD80, and CD86; myeloid markers, such as CD11b and CD11c (Figure 1A). Our results show that human monocytes cultured *in vitro* spontaneously develop into a monocytic–macrophage phenotype as observed by the expression of CD14, CD163, CD11b, and CD11c, but that they also express co-stimulatory molecules, such as CD86 and HLA-DR. In comparison, cells cultured in BEC-CM, in addition to the previous markers, noticeably increased the expression of CD141, CD123, and DC-SIGN, markers that have been identified in different subpopulations of DCs, thus suggesting that this particular phenotype may identify a distinctive cell type.

### BEC-CM, as well as Primary Alveolar- or Nasal Epithelial Cell-Conditioned Media Modulated the Surface Phenotype of Monocytes

In order to investigate whether the CD141/CD123/CD-SIGN phenotype was also detectable in DCs, monocytes were cultured in GM-CSF (10 ng/mL) and IL-4 (10 ng/mL) to obtain DCs. Figures 1B–D show that BEC-CM significantly increased the expression of CD141, CD123, and DC-SIGN in monocytes, in contrast to DCs where no upregulation of any of these receptors was observed, but rather a decreased constitutive expression of DC-SIGN in immature DCs, suggesting that the modulation of the epithelial environment is specific to monocytes. To confirm the role of the epithelial environment, CM obtained from primary nasal epithelial cells (PNECs) or primary alveolar epithelial cells (PAECs) were compared with monocytes cultured in their respective epithelial media. As shown in Figures 1E–G, BEC-CM significantly increased the expression of CD141, CD123, and DC-SIGN. PAEC-CM has been shown to increase the expression of CD141 and CD123. In contrast, PNEC-CM only increased CD141 expression, suggesting that CD123 is a receptor that may play a role in the airways within the bronchial or alveolar environment. DC-SIGN seems to be a receptor only modulated by the bronchial environment. In contrast, CD141 seems to be a receptor modulated by the epithelial environment regardless of the epithelial origin. As a control, monocytes were cultured in BEC-CM obtained from primary lung endothelial cells or fibroblasts, and none of these conditions upregulated the expression of these receptors as shown in Figure S1 in Supplementary Material. These results confirm that the epithelium specifically modulates a CD141 phenotype in monocytes and is not restricted to the epithelial BEAS 2B cell line. CD123/CD-SIGN modulation may depend on the local environment.

### BEC-CM Upregulates Expression of *FLT3* in Monocytes

**Figure 2:**
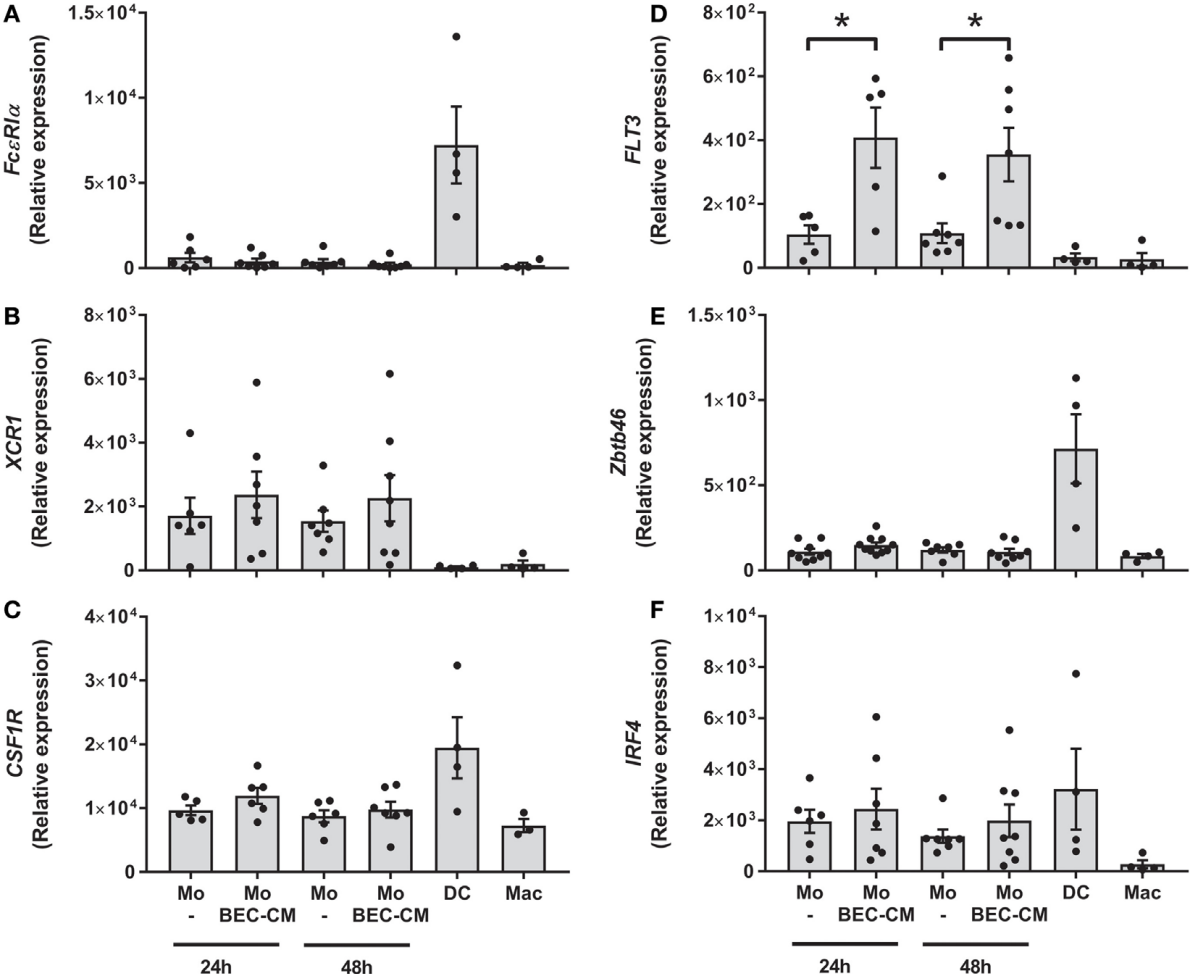
**Expression of monocyte-derived DC markers in bronchial epithelial cell-conditioned monocytes**. Monocytes were cultured for 24 or 48 h with medium alone or with Bronchial epithelial cell-conditioned media (BEC-CM). Gene expression of **(A)** *Fc*ε*R1*α, **(B)** *XCR1*, **(C)** *CSF1R*, **(D)** *FLT3*, **(E)** *ZBTB46* and **(F)** *IRF4* were assessed by the real-time quantitative PCR. Data express the relative gene abundance normalize to the endogenous control β-2-microglobulin (B2M) and are shown as mean ± SEM of four to six independent experiments, done in duplicates. Statistical analysis was performed using analysis of variance test (followed by Tukey multiple comparisons test) (*P < 0.05).

To address whether BEC-conditioned monocytes are related to ModDCs, we analyzed the expression of a group of genes involved in DC and ModDC development (22, 31, 32), which includes *Fc*ε*R1*α, *XCR1, CSF1R, FLT3, IRF4*, and *ZBTB46* (Figures 2A–F). Monocytes were cultured with medium alone or with BEC-CM for 24 or 48 h. For comparative purposes, we also analyzed the gene expression in DCs or macrophages. Consistent with previous reports, DCs expressed the highest levels of the transcription factors, *ZBTB46* and *IRF4*, and the receptors, *Fc*ε*R1*α and *CSF1R* (Figures 2A,C,E,F). Monocytes expressed high levels of *XCR1* and intermediate levels of *CSF1R* and *IRF4*, compared to DCs. However, their expression was not further increased by BEC-CM (Figures 2B,C,F). Importantly, BEC-CM upregulated the expression of *FLT3* and the modulation was already high at 24 h (Figure 2D). In contrast, macrophages only expressed *CSF1R*, a growth factor receptor involved in macrophage development (Figure 2C). These results suggest that monocyte development closely resembles that of DCs. The fact that BEC-CM is able to increase the expression of *FLT3*, a receptor involved in DC differentiation and maintenance in the periphery (33), suggests that the bronchial epithelium participates in the transition of monocytes toward a ModDC phenotype.

### BEC-CM Stimulated the Secretion of IL-1β, IL-6, and IL-10 by Monocytes

**Figure 3:**
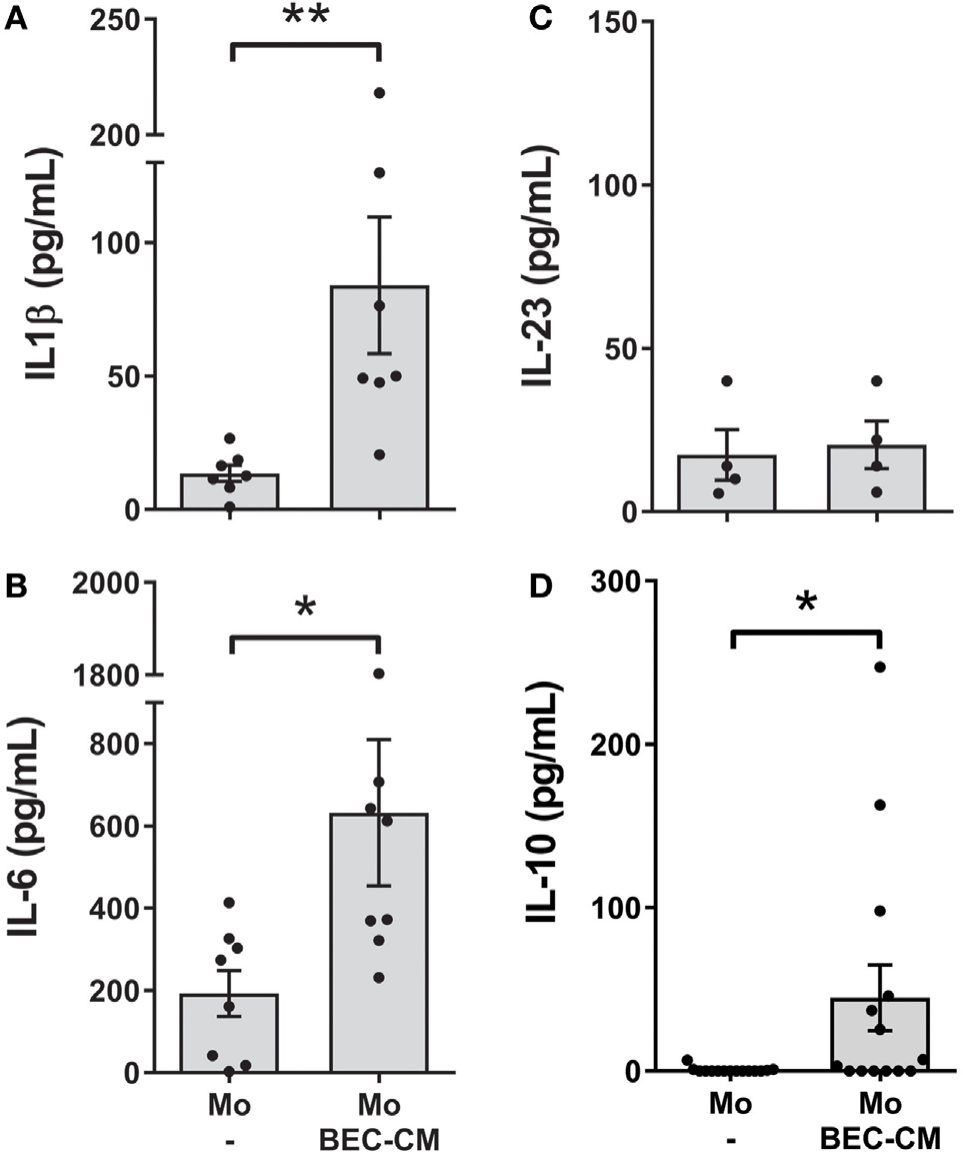
**Bronchial epithelial cell-conditioned media (BEC-CM) stimulates the secretion of IL1β, IL-6, and IL-10 in monocytes**. Monocytes were cultured for 48 h with medium alone or with BEC-CM. The secretion of cytokines was analyzed using the Luminex system or ELISA: **(A)** IL-1β, **(B)** IL-6, **(C)** IL-23, and **(D)** IL-10. Data are expressed as means ± SEM of at least four independent experiments. Statistical analysis was performed using Mann–Whitney test (*P < 0.05, **P < 0.01).

Next, to investigate whether endogenous molecules produced by BEC-CM were capable of activating monocytes, IL-10, IL-6, IL-1β, IL-23, IL-12p70, and TNF-α were analyzed after 48 h of culturing monocytes in BEC-CM. Indeed, as show in Figures 3A–D, BEC-CM induced the release of IL-1β, IL-6, and IL-10 by monocytes. The observed release of cytokines corresponds to the cytokines released by monocytes because, as shown in Figure S2 in Supplementary Material, in basal culture conditions, BECs did not release IL-1β, IL-10, and lower levels of IL-6. The release of IL-12p70 and TNF-α were not modulated by BEC-CM (data not shown). These results suggest that endogenous molecules released constitutively by BECs are already sufficient in order to induce specific phenotypic and functional modifications of monocytes.

### BEC-Conditioned Monocytes Efficiently Trigger the Differentiation of Human Th17 Cells through a Mechanism Involving IL-1β, but Also Stimulate Memory Cells to Produce IL-10

**Figure 4:**
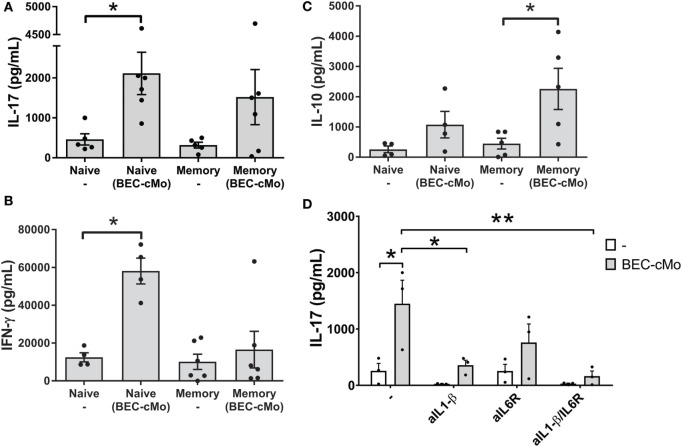
**Monocytes cultured in bronchial epithelial cell-conditioned media (BEC-CM) have an increased ability to trigger the differentiation of human naive T cells into IL-17 producers**. Allogenic Naive T cells or Memory T cells were stimulated with conditioned monocytes [cultured with medium alone or with BEC-CM (BEC-cMo)] at a monocyte:T-cell ratio of 1:10 for 9 days, supplemented with IL-2 for the last 2 days and then stimulated for 5 h with PMA and ionomycin. Supernatants were analyzed for the release of **(A)** IL-17, **(B)** IFN-γ, and **(C)** IL-10. Data are expressed as means ± SEM of four to six independent experiments. Statistical analysis was performed using a Mann–Whitney test (*P < 0.05). **(D)** Production of IL-17 by naive T-cells primed with allogenic monocytes that were cultured with medium alone or with BEC-CM at a monocytes:T-cell ratio of 1:10. During the mixed lymphocyte reaction, cells were cultured in the presence of neutralizing antibodies to IL-1β, IL-6R, or both. Data are expressed as mean ± SEM of three independent experiments. Statistical analysis was performed using analysis of variance test (followed by Tukey multiple comparisons test) (*P < 0.05, **P < 0.01).

Based on the observation that monocytes cultured in BEC-CM released pro-Th17 cytokines, such as IL-1β and IL-6, we asked whether or not BEC-conditioned monocytes were capable of priming an inflammatory response, such as Th17 or Th1 responses or of stimulating memory cells to produce IL-10. For these experiments, monocytes were cultured with medium alone or with BEC-CM for 48 h. Subsequently, cells were washed and then cocultured with allogenic naive or memory T cells for 7 days and then supplemented with IL-2 for two additional days. During the co-culture, monocytes cultured in BEC-CM were able to increase the release of IL-17 in naive T-cells while maintaining a strong Th1 response through the release of IFN-γ (Figures 4A,B). This result contrasts with that of memory T cells, in which BEC-conditioned monocytes mostly favored the induction of an anti-inflammatory response as measured by the release of IL-10 (Figure 4C). BEC-conditioned monocytes to some extent induced the release of IL-17, albeit not significantly. To further document the function of BEC-CM, and hence the role of IL-1β and IL-6 in the ability of BEC-CM-monocytes to polarize naive T cells into Th17 cells, we evaluated the effects of anti-IL-1β and anti-IL-6 receptor (IL-6R) neutralizing antibodies during the allo-reaction. Treating IL-1β or IL-1β and IL-6R with neutralizing antibodies abolished the release of IL-17 by naive T-cells (Figure 4D). These results indicate that the ability of monocytes cultured in BEC-CM to induce IL-17 release is critically dependent on the production of IL-1β and on the synergistic effect of IL-6 with IL-1β, but is not dependent on IL-6 alone. Overall, these results demonstrate that BEC-CM has an important capacity to modulate monocyte function, resulting in distinct T-helper responses, such as a strong inflammatory response when the monocytes encounter a naive T cell, but a more anti-inflammatory response when they encounter a memory T cell.

### BEC-CM Differentially Regulates the Release of Cytokines by Monocytes and DCs after LPS Treatment

**Figure 5:**
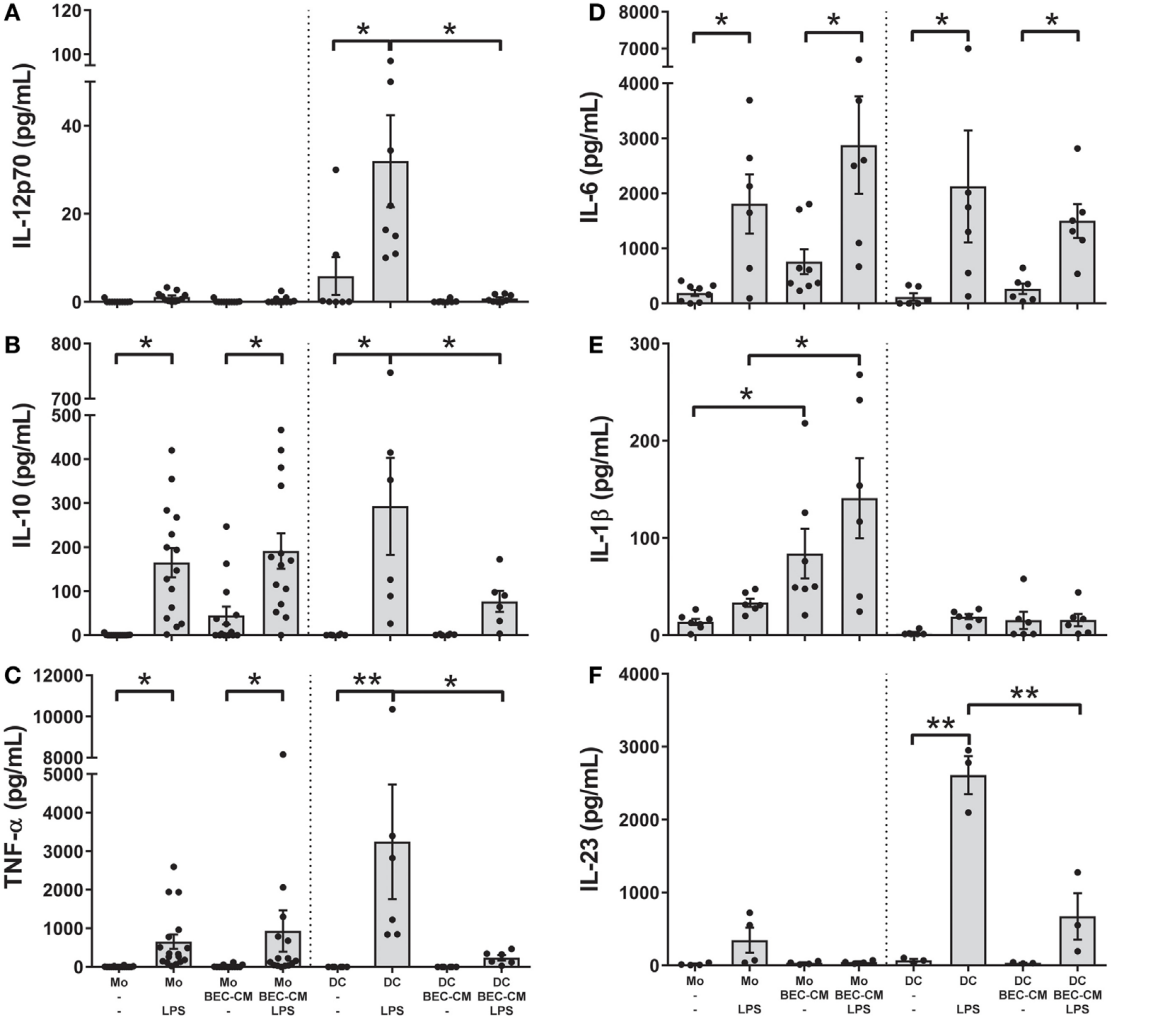
**Bronchial epithelial cell-conditioned media (BEC-CM) regulates the release of cytokines by monocytes and dendritic cells (DCs) differentially after lipopolysaccharide (LPS)**. Monocytes and DCs were cultured for 48 h with medium alone or with BEC-CM. Cells were stimulated with LPS (100 ng/mL) for the last 24 h. The secretion of cytokines was analyzed using the Luminex system or ELISA: **(A)** IL-12p70, **(B)** IL-10, **(C)** TNF-α, **(D)** IL-6, **(E)** IL-1β, and **(F)** IL-23. Data are expressed as means ± SEM of at least four independent experiments. Statistical analysis was performed using analysis of variance test (followed by the Fisher LSD test) (*P < 0.05, **P < 0.01).

To better characterize the role of the epithelium-soluble components on monocyte function, monocytes and DCs were cultured with medium alone or with BEC-CM for 24 h and further stimulated with LPS (100 ng/mL). The release of the cytokines IL-12p70, IL-10, TNF-α, IL-6, IL-1β, and IL-23 was analyzed after 24 h of LPS stimulation. As shown in Figures 5A–F, monocytes stimulated by LPS released IL-10, TNF-α, and IL-6, but no significant amounts of IL-1β or IL-23 and no IL-12p70 at all. BEC-conditioned monocytes released IL-10 and TNF-α after LPS stimulation as did the monocytes alone. As mentioned previously, BEC-conditioned monocytes are able to produce IL-6, IL-1β, and IL-10, but upon LPS stimulation only the release of IL-6 and IL-10 were further increased (Figures 5B,D,E). In regard to DCs and in line with previously published results (5, 34), BEC-CM abrogated the release of the inflammatory cytokines IL-12p70, IL-10, TNF-α, and IL-23 and caused a partial reduction of IL-6 in LPS-stimulated DCs. Overall, these results highlight the distinct inflammatory response induced by the epithelial-conditioned environment which promotes an inflammatory state in monocytes, but maintains a strong immunosuppression on DCs.

### Ligation of CD141 on Monocytes in the BEC-CM Environment Suppresses the Release of TNF-α when Stimulated by LPS

**Figure 6:**
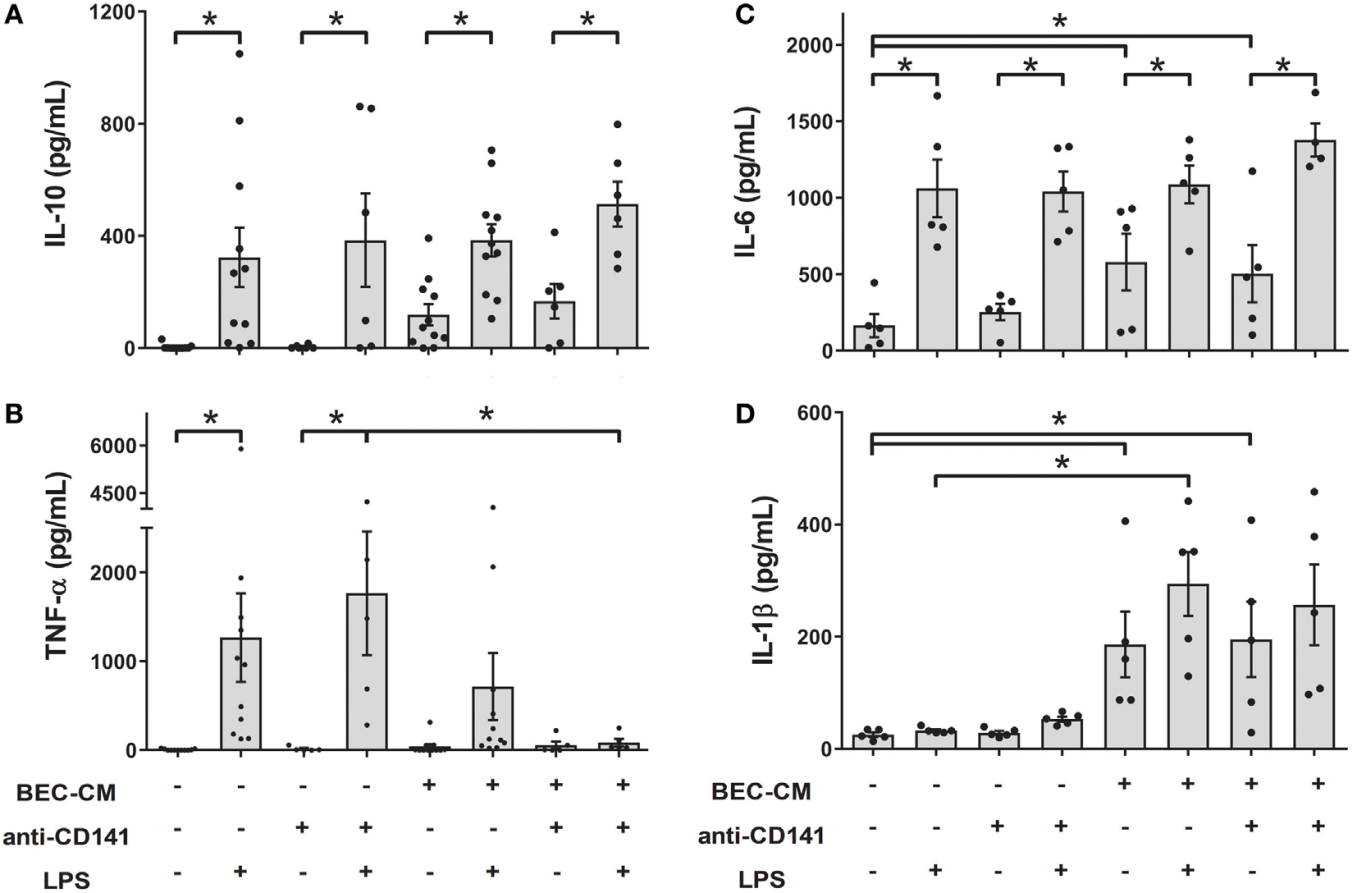
**Ligation of CD141 in the bronchial epithelial cell-conditioned media (BEC-CM) environment suppresses the release of TNF-α when stimulated by lipopolysaccharide (LPS)**. Monocytes were cultured for 48 h with medium alone or with BEC-CM in the presence of the anti-CD141 antibody (0.5 μg/mL) or 0.5 μg/mL mouse IgG1 isotype control. Cells were stimulated with LPS (100 ng/mL) for the last 24 h. The secretion of cytokines was analyzed using the Luminex system: **(A)** IL-10, **(B)** TNF-α, **(C)** IL-6, and **(D)** IL-1β. Data are expressed as means ± SEM of at least five independent experiments. Statistical analysis was performed using analysis of variance test (followed by the Fisher LSD test) (*P < 0.05).

As shown in Figure 1B, the CD141 receptor appears to be specifically modulated by BEC-CM. Thus, in order to understand the role of CD141 in the monocyte inflammatory response, monocytes were cultured with medium alone or with BEC-CM in the presence or absence of anti-CD141 for 24 h and were further stimulated for 24 h with LPS. As shown in Figure 6, ligation of CD141 did not significantly modulate the LPS-induced release of IL-10, IL-6, or IL-1β in cells cultured in media alone or BEC-CM (Figures 6A–D). However, ligation of CD141 specifically abrogated the release of TNF-α only in the BEC environment (Figure 6B), suggesting that CD141 downstream signaling events impact LPS-induced TNF-α expression or release, and this pathway can be blocked by the synergistic effect of CD141 activation and epithelial soluble components.

### Inflammatory Cytokines Boosted the Expression of CD141 in Monocytes Cultured in BEC-CM, but Do Not Modulate *FLT3* Expression

**Figure 7:**
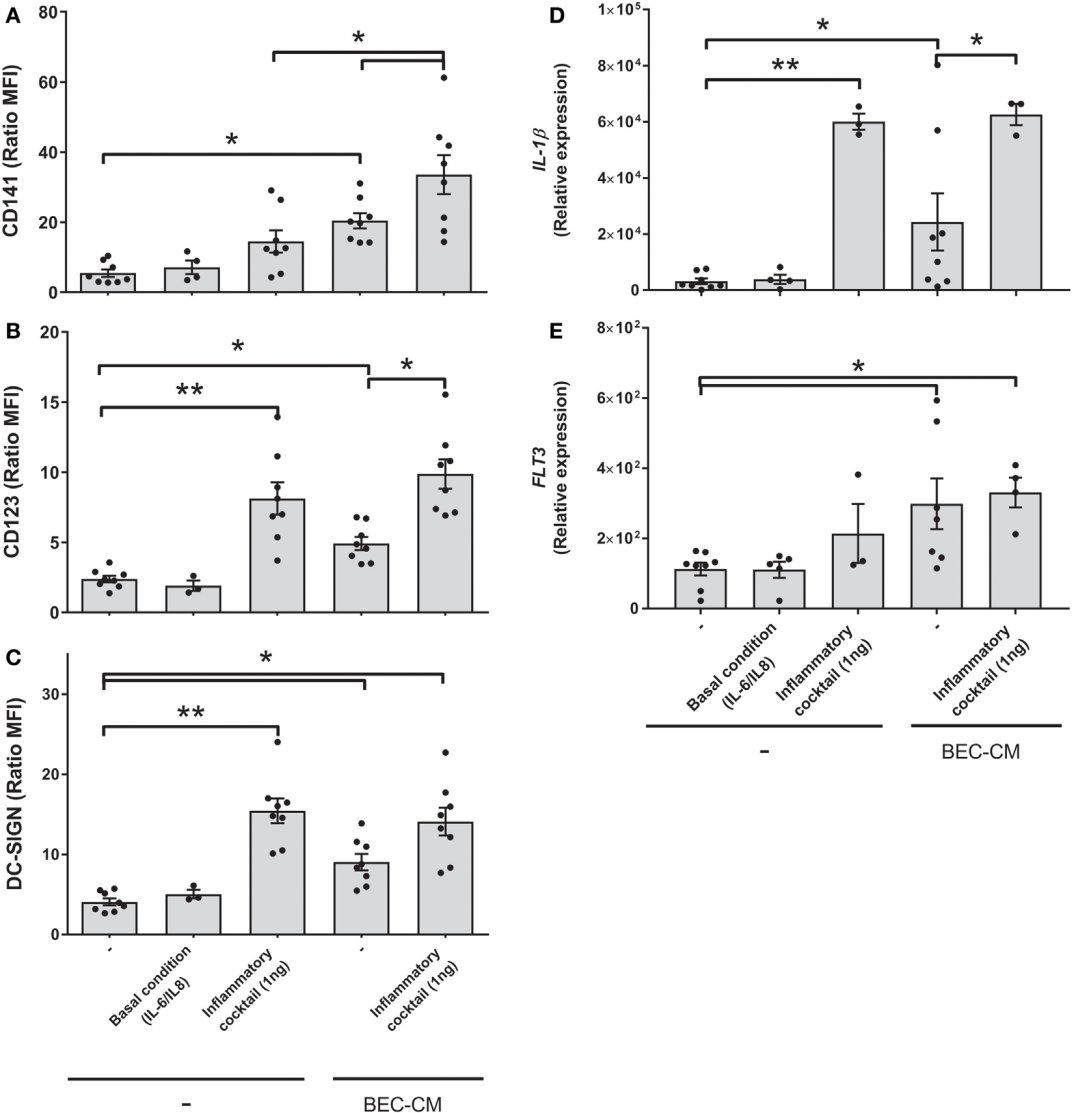
**Inflammatory cytokines boosted the expression of CD141 in monocytes cultured in bronchial epithelial cell-conditioned media (BEC-CM)**. Monocytes were cultured for 48 h **(A–C)** or 24 h **(D,E)** with medium alone or with BEC-CM. Cells were supplemented with IL-6 (100 pg/mL) and IL-8 (500 pg/mL) designated as the basal condition or with 1 ng/mL of the following cytokines: IL-6, IL-8, IL-15, IL-1β, GM-CSF, and TNF-α, designated as the inflammatory cocktail. The mean fluorescence intensity ratio calculated with its specific isotype for the expression of **(A)** CD141, **(B)** CD123, and **(C)** DC-SIGN is plotted. Data are expressed as means ± SEM of eight independent experiments (*P < 0.05). Statistical analysis was performed using analysis of variance test (ANOVA) (followed by Tukey multiple comparisons test). Gene expression of **(D)** *IL-1*β and **(E)** *FLT3* were assessed by real-time quantitative PCR. Data express the relative gene abundance normalized to the endogenous control β-2-microglobulin (B2M) and are shown as mean ± SEM of three to six independent experiments, performed in duplicate. Statistical analysis was performed using ANOVA (followed by Tukey multiple comparisons test) (*P < 0.05, **P < 0.01).

With the aim to mimic sterile inflammation and hence determine whether inflammatory cytokines directly modulate the CD141/CD123/DC-SIGN phenotype and expression of *IL-1*β and *FLT3*, monocytes were cultured with medium alone or with BEC-CM supplemented with IL-6 (100 pg/mL) and IL-8 (500 pg/mL) at concentrations that were found to be produced by ECs in basal culture conditions (Figure S2 in Supplementary Material) or cultured with an inflammatory cocktail composed of 1 ng/mL of IL-6, IL-8, IL-1β, IL-15, TNF-α, and GM-CSF cytokines produced by ECs during inflammation (35). As shown in Figures 7A–E, IL-6 and IL-8, in basal conditions, were not sufficient to induce the modulation of the CD141/CD123/DC-SIGN phenotype or the *IL-1*β and *FLT3* expression on monocytes. In contrast, the inflammatory cocktail modulated the expression of DC-SIGN and CD123 in monocytes and CD123 expression was further increased by BEC-CM (Figure 7B). CD141 receptors on monocytes showed a different modulation. CD141 was not significantly upregulated by inflammatory cytokines in monocytes alone, but the expression of CD141 was boosted in monocytes cultured in BEC-CM (Figure 7A). Regarding *FLT3* and *IL-1*β expression, both were significantly modulated by the BEC-CM. The inflammatory cocktail was sufficient to modulate the expression of *IL-1*β in monocytes, although *FLT3* expression is independent of the inflammatory conditions. Overall, these results highlight how endogenous molecules, such as cytokines, differentially regulate the phenotype and function of monocytes in which CD123, DC-SIGN, and IL-1β showed to be directly modulated by inflammation. Of particular interest, modulation of CD141 seems to require a synergistic effect between cytokines and other endogenous molecules produced by BECs and *FLT3*, which are not modulated explicitly by inflammation. None of the other ModDC genes were modulated by inflammatory cytokines (data not shown).

### The CD141/CD123/DC-SIGN Phenotype Can Be Found in the BALF of Patients with Sarcoidosis

**Figure 8:**
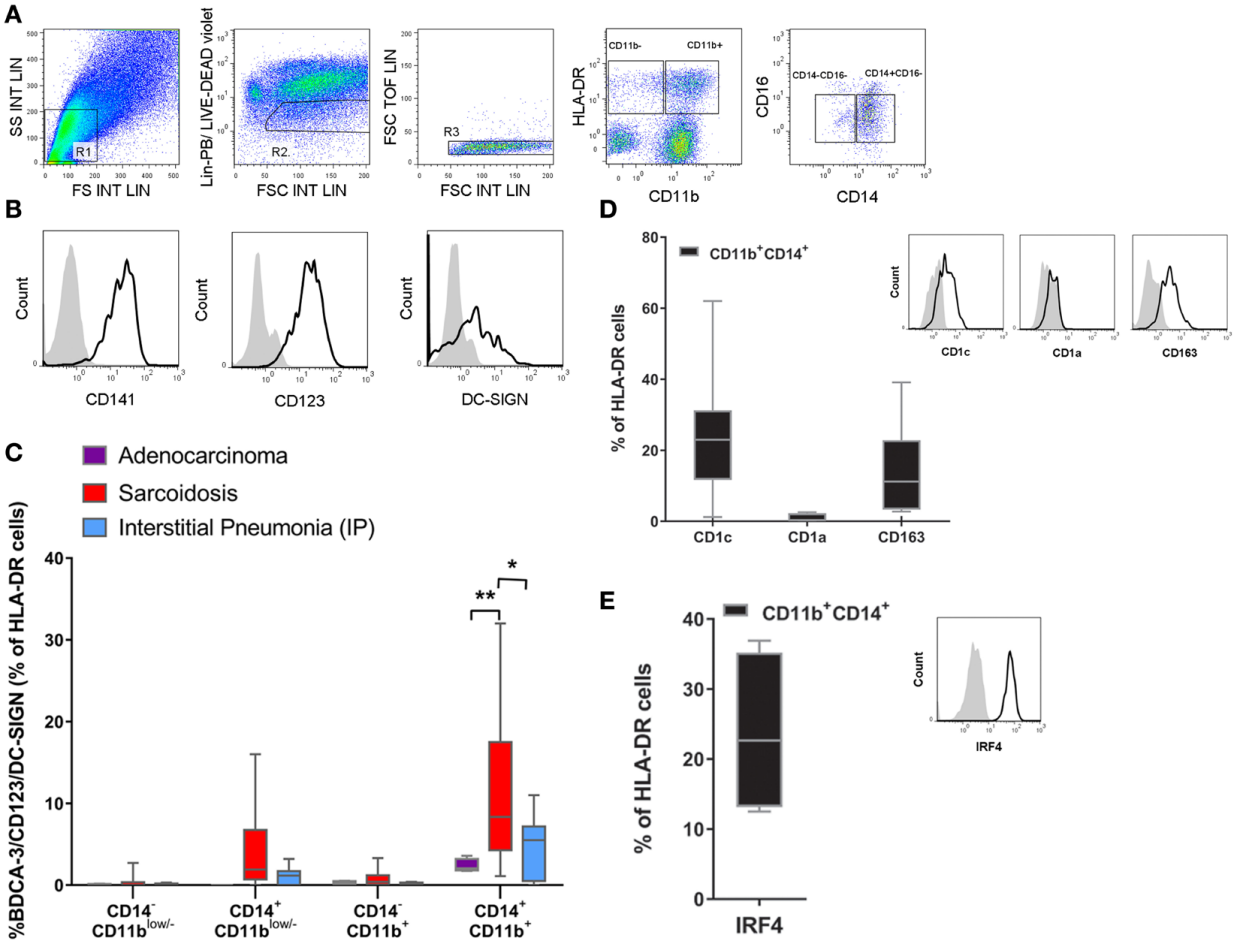
**Identification of the CD141/CD123/DC-SIGN phenotype in bronchoalveolar lavage fluid (BALF)**. Cells from BALF were obtained from patients with lung adenocarcinoma (*n* = 4), sarcoidosis (*n* = 13), or interstitial pneumonia (*n* = 7). Cells were stained with anti-lineage (Lin) (CD3, CD19, CD 56), HLA-DR, CD11b, CD14, CD16, CD141, CD123, and DC-SIGN antibodies and analyzed by flow cytometry. **(A,B)** One representative experiment out of 13 samples from sarcoidosis patients is shown. Total cells were gated as depicted. From the FSC/SSC R1 gate, cells were gated into lin^−^ and live cells (R2) and doublets exclusion (R3). Then cells were gated by HLA-DR expression and further selected by CD11b expression. **(B)** Histograms display the expression of the surface molecules CD141, CD123, and DC-SIGN of the gated population Lin^−^, HLA-DR^+^, CD11b^+^, CD14^+^, and CD16^−^ in a sarcoidosis patient compared with the corresponding isotype control. **(C)** Percentage of the triple-positive CD141/CD123/DC-SIGN population among Lin^−^ HLA-DR^+^ and subsequent analysis in CD11b and CD14/CD16 gates. Data are depicted in box-and-whisker graph (*P < 0.05, **P < 0.01). Statistical analysis was performed using Two-way analysis of variance test (followed by Tukey multiple comparisons test). **(D,E)** Cell percentage and representative histograms of CD1c, CD1a, CD163, and intracellular IRF4 in the Lin^−^ HLA-DR^+^CD11b^+^CD14^+^CD16^−^ population. Data are expressed as means ± SEM of at least four BALF with sarcoidosis.

In order to determine whether the expression of CD141/CD123/DC-SIGN is biologically significant, and hence to identify a specific population in the lung, we analyzed cells from the BALF of patients with two distinct lung diseases: sarcoidosis and interstitial pneumonia. Furthermore, we had the opportunity to analyze some BALF from patients who had developed adenocarcinoma in which no clinical inflammation or development of infection could be found. Cells obtained from BALF were stained using a combination of phenotypic markers found on monocytes, macrophages, and DCs (Figures 8A–E). After the exclusion of cells gated as lin^−^ (R2) and doublets (R3), live cells were gated by HLA-DR expression and further selected by their CD11b expression with the aim of differentiating classical DCs from monocytes. Then, cells were further selected by their expression of CD14^+^ and CD16^+^, and we evaluated the expression of the CD141/CD123/DC-SIGN triple-positive cell in each of the four gates generated. In Figure 8B, expression of the surface molecules CD141, CD123, and DC-SIGN is depicted. It is worth mentioning that the frequency of CD11b and CD14/CD16 cells was similar in all patients (Figure S3 in Supplementary Material). As we can observe in Figure 8C, a triple-positive population can be found in the HLA-DR^+^, CD11b^+^, CD14^+^, CD16^−^ gate in all patients. It was expressed at low frequency (2.3% mean ± 0.4 SEM) in patients with adenocarcinoma, slightly increased in patients who had developed lung fibrosis (4.6% mean ± 1.5 SEM), and was significantly increased in patients with pulmonary sarcoidosis (11.2% mean ± 2.4 SEM), which is a disease with a lymphocytic inflammatory pattern (28). This is a key observation as it demonstrates that the CD141/CD123/DC-SIGN population can be associated with inflammation and may play a role in the pathogenesis of sarcoidosis. Moreover, as shown in Figures 8D,E, this population also expresses CD1c and CD163, but not CD1a, and additionally stained positive for IRF-4, a transcriptional factor expressed in *bona fide* migratory DCs. Overall, these results suggest that the population expressing CD141/CD123/DC-SIGN resembles monocyte-derived DCs and, in sarcoidosis, may be a subset related to inflammatory DCs.

## Discussion

Little is known about the role of the epithelium modulating monocyte function upon arrival in the lung parenchyma. In the present study, we demonstrate that bronchial epithelial conditioned media (BEC-CM) favors, on monocytes, the release of IL-10 and IL-6, but particularly the expression and release of IL-1β, thus resulting in the induction of naive Th17 cells and IL-10-producer memory T cells. This particular phenotype is characterized by the surface expression of CD141/CD123/DC-SIGN and *FLT3* gene expression and may represent ModDCs.

Our results show that monocytes express *XCR1, IRF4*, and *CSF1R*, molecular features described in ModDC development as part of the DC and macrophage signatures. However, their expression is not further increased by BEC-CM. *FLT3* expression is low in monocytes, whereas it is upregulated by BEC-CM, thus establishing a close link to DC development, since it has been demonstrated that FLT3L is a pivotal growth factor in the maintenance of DC homeostasis in peripheral lymphoid tissues in the steady state (36). *ZBTB46* and *Fc*ε*RI*α gene expression was not modulated by BEC-CM, and none of the genes analyzed were significantly modulated by inflammatory cytokines. This suggests that to fully commit monocytes to become inflammatory DCs, they may require additional signals.

In our study, IL-1β played a key role. It was surprising that BEC-CM, without microbial stimulus, was capable of inducing the release of IL-1β by monocytes but not by DCs. BEC-CM induced the expression and release of IL-1β, thus supporting a one-step model for IL-1β secretion. Recent studies demonstrated that, in human monocytes, IL-1β can be secreted by an “alternative inflammasome pathway,” which is distinct from classical inflammasome activation and does not require a second stimulus (37). Moreover, it has been reported that a variety of endogenous molecules can also signal, through pattern recognition receptors, the induction of IL-1β secretion (38). In view of these results, we hypothesized that cytokines released by ECs are at the origin of the expression and release of IL-1β. As we reported previously (35) and as is shown in Figure S2 in Supplementary Material, BECs constitutively release cytokines and chemokines, which constitute basal level conditions. Our results show that IL-6 and IL-8, the major cytokine components of the basal culture condition, were not sufficient to modify the expression of IL-1β on monocytes. In future experiments, we will analyze other components of ECs which are known to have immune regulatory functions, such as nitric oxide, and lipid mediators, such as prostaglandin-E2 (6, 39, 40).

Our *in vitro* experiments show that BEC-conditioned monocytes are also capable of expanding both naive and memory human T helper cells, although each with a different phenotype. On one hand, BEC-conditioned monocytes were able to polarize naive CD4 T cells toward Th1/Th17 cells with a low level of IL-10 production. On the other hand, BEC-conditioned monocytes were also able to expand memory CD4 T cells that had a regulatory phenotype instead by the marked production of IL-10 and to some extent, albeit not significantly, IL-17. There is increasing evidence about the existence of pathogenic and non-pathogenic Th17 (41). It has been reported that the cellular response to IL-23, not IL-17 itself, is the major factor driving pathogenic Th17 (42). The fact that IL-23 is not a cytokine produced by BEC-conditioned monocytes even upon LPS stimulation, leads us to believe that, in tissues where a BEC-conditioned monocyte encounters a memory T cell, it will have a specific role in homeostatic immune function and if there is any IL-17 induced it will then be more protective, e.g., against a fungal infection. Nevertheless, BEC-conditioned monocytes are able to prime naive T cells into a more inflammatory IL-17/IFN-γ phenotype in which only the inhibition of IL-6 in combination with IL-1β, or of IL-1β alone, were critically important for the expansion of Th17. To what extent the IL-17 induced by BEC-conditioned monocytes in basal conditions or during inflammation is pathogenic or non-pathogenic will be explored in future studies.

Another important aspect of our studies is the demonstration of the different specificities of BEC-CM on activation of monocytes and DCs. DCs cultured in BEC-CM developed an anti-inflammatory phenotype where the release of IL-12p70, IL-23, TNF-α, and IL-10 upon LPS stimulation was impaired. These results are largely supported by previous studies in which the epithelial environment generated by lung or intestinal ECs generates DCs with regulatory features (4–6, 43). In contrast, upon LPS stimulation, BEC-conditioned monocytes preserved the release of cytokines, thus maintaining the innate inflammatory response. Overall, our data depict a distinct, cell-specific modulation and regulatory function by BEC-CM on monocytes and DCs.

The functions of CD123 and DC-SIGN have been well documented in the past few years (44, 45); however, the role that the thrombomodulin receptor CD141 may play in the context of monocyte and DC modulation remains unknown. In our experiments, CD141 is a receptor shown to be upregulated by ECs localized in the lower or upper respiratory tract. Ligation of CD141 on monocytes in the bronchial epithelial environment blocked the LPS-mediated release of TNF-α, suggesting that this receptor may be involved in the inhibition of a TNF-inflammatory axis during LPS inflammation. However, CD141 did not show any modulation upon LPS stimulation (data not shown). In contrast to CD141, DC-SIGN, a pattern recognition receptor, was not modulated by PNEC-CM or PAEC-CM. This result was unexpected with PAEC-CM since the alveolar environment is a key location for the neutralization of invading pathogens. Our result is supported, however, by previous studies, which showed that monocytes cultured in CM, from the alveolar cell line A-549 from mock or respiratory syncytial virus CM, do not upregulate this receptor (35). In future studies, we will explore to what extent alveolar ECs specifically modulate DC-SIGN expression in macrophages but not in monocytes (46).

Over the past decades, the host immune response to microorganisms has been extensively studied. However, for several chronic lung diseases, such as sarcoidosis and idiopathic pulmonary fibrosis, for which the etiology and pathogenesis are still unknown, endogenous signals, such as cytokines, growth factors, proteases and, in general, damage-associated molecular pattern molecules resulting from recurrent injury, are highly correlated to the loss of lung function and in some cases to the maintenance of chronic inflammation (47, 48). To gain insight into the modulation of BEC-conditioned monocytes during sterile inflammation, BEC-CM was supplemented with inflammatory cytokines. Inflammatory cytokines modulated the expression of CD141/CD123/DC-SIGN receptors with different kinetics. They sufficiently upregulated CD123 and DC-SIGN receptors in monocytes, but not CD141. Indeed, a synergistic effect was observed for CD141 expression as it was particularly upregulated in the epithelial environment, suggesting that inflammatory cytokines with unknown endogenous signals have a synergistic effect on CD141 expression. Based on our results, CD141/CD123/DC-SIGN is a phenotype modulated by BEC-CM under basal culture conditions. Moreover these receptors were further upregulated during sterile inflammation and they were also shown to be upregulated after viral-infected BEC-CM (Figure S4 in Supplementary Material). We hypothesized that during inflammation the frequency of the CD141/CD123/DC-SIGN population increases *in vivo*.

Thus, we addressed whether this specific CD141/CD123/DC-SIGN phenotype can be found *in vivo* and whether this phenotype correlates with an inflammatory state. Multicolor flow cytometry was performed in cells obtained from the BALF of patients with different lung diseases. As expected, we found CD141/CD123/DC-SIGN triple-positive monocytes in the HLA-DR^+^CD11b^+^CD14^+^D16^−^ fraction. The fact that the triple-positive population was present at low frequency (2.3% mean ± 0.4 SEM) in patients with adenocarcinoma, slightly increased in patients who had developed a lung fibrosis (4.6% mean ± 1.5 SEM), and significantly increased in patients with pulmonary sarcoidosis (11.2% mean ± 2.4 SEM) suggests that the frequency of this phenotype could be related to inflammation, since in patients who had developed adenocarcinoma, there were no signs of clinical inflammation or infection. In regard to the fibrotic environment, which is characterized by the expression of fibrotic cytokines, e.g., TGFβ-1 (49, 50), the CD141/CD123/DC-SIGN population seems not to be significantly involved. However, sarcoidosis, a disease characterized by the formation of granulomas and increased numbers of lymphocytes and inflammatory mediators, such as IL-12 and TNF-α but also IL-10 (28, 48, 51), was associated with an increased frequency of the CD141/CD123/DC-SIGN population. Further investigation is required in order to determine the role that this population can play in each of the lung diseases, particularly in inflammation. Furthermore, it is essential to determine the role of the epithelium *in vivo* and whether or not a dysregulated function is responsible for the accumulation of this particular population during inflammation.

It has been very recently described that a ModDC subset may exist in humans (21, 22, 52, 53). Two recent papers have identified a particular subset of monocytes expressing HLA-DR^+^CD14^+^CD16^−^ CD206^+^CD1c^+^ with variable levels of CD1a in BALF and lung tissues at steady state (21). Baharom et al. described a similar subset of ModDCs on the basis of *IRF4* expression (53). However, there is disagreement in regard to the expression of CD141. Baharom et al. mentioned that this HLA-DR^+^CD14^+^CD16^−^CD206^+^ population also upregulates CD141. In contrast, Desch et al. classified the HLA-DR^+^CD14^+^CD141^+^DC-SIGN^+^ population as tissue monocytes. This discrepancy may be due to differences in gate strategy since Desch et al. included both CD16^high^ and CD16^−/low^ fractions in their analysis. Our analysis demonstrated that the CD141/CD123/DC-SIGN population in the HLA-DR^+^ CD14^+^ CD16^−^ fraction also expresses CD1c. Whether the CD14^+^CD1c^+^ (HLA-DR^+^CD16^−^CD206^+^) population, as described by previous reports at steady state and the CD141/CD123/DC-SIGN (HLA-DR^+^CD14^+^ CD16^−^CD1c^+^D1a^−^) population, as reported in this study, are different subsets or are cells with the same origin but in different phases of development (from steady state to inflammation), has to be further studied. What is noteworthy and highlights the significance of the correlation of our triple-positive cells with ModDCs, is that at steady state in axillary and pulmonary lymph nodes (LNs), in addition to the classical DCs (pDCs, CD141-DCs, CD1c-DCs CD1a and Langerhans), one additional population was identified: the CD141^+^CD14^+^ population, which also expresses HLA-DR^+^CD11c^+^CD206^+^ (21, 52). The studies performed in axillary LN reported that this population also expresses DC-SIGN and suggest that it is a migratory skin DC subset (52). We speculate that the CD141/CD123/DC-SIGN triple population might be a migratory ModDC subset, on the basis of the phenotype expressed, but also, based on its expression of *IRF4*, a transcriptional factor, which is known to play a role in the migration of peripheral tissue DCs (31).

In summary, our results contribute to the understanding of the mechanism by which BECs modulate local immune responses. Soluble components of the epithelium promoted a particular CD141/CD123/DC-SIGN phenotype in monocytes thus inducing the expression of the *FLT3* gene making this phenotype resemble that of ModDCs. Functionally, BEC-conditioned monocytes, by releasing IL-6, IL-1β, and IL-10, are capable of inducing inflammatory Th17 responses, but also capable of inducing IL-10 producing memory T cells. This highlights the specific immune regulation induced by the epithelium. The frequency of the CD141/CD123/DC-SIGN population is specifically increased in patients with sarcoidosis, an inflammatory lung disorder. Further investigation is needed in order to determine which epithelial endogenous signals are responsible for the induction of this particular phenotype and in order to decipher the mechanisms by which the CD141/CD123/DC-SIGN/*FLT3* monocytes tip the balance in favor of a pathogenic or non-pathogenic response.

## Ethics Statement

PBMCs were isolated from the buffy coats of healthy donors. In accordance with the Cantonal Ethics Committee of the Canton of Vaud (Vaud-Switzerland), written consent from the donors was obtained from the Lausanne blood transfusion center. Human primary nasal epithelial cells (PNECs) were obtained from healthy volunteers. This study was carried out in accordance with the recommendations of the Cantonal Ethics Committee of the Canton of Bern, with written informed consent from all subjects. All subjects gave written informed consent in accordance with the Declaration of Helsinki. The protocol was approved by the Cantonal Ethics Committee of the Canton of Bern (Bern-Switzerland No. 1104). Human primary alveolar epithelial cells (PAECs) were cultured from the tissue of patients undergoing surgical lung resection due to lung cancer. This study was carried out in accordance with the recommendations of the Human Ethics Committee of Basel with written informed consent from all subjects. All subjects gave written informed consent in accordance with the Declaration of Helsinki. The protocol was approved by The Human Ethics Committee of Basel (EKBB # 05/06, Department of Pneumology, University of Basel, Switzerland).

Bronchoalveolar lavage fluid samples were obtained from patients undergoing bronchoscopy for medical reasons by the CHUV Pneumology Service. This study was carried out in accordance with the recommendations of the Cantonal Ethics Committee of the Canton of Vaud with written informed consent from all subjects. All subjects gave written informed consent in accordance with the Declaration of Helsinki. The protocol was approved by the Cantonal Ethics Committee of the Canton of Vaud (Vaud-Switzerland/ protocol 294/1).

## Author Contributions

AG, FB: study design, performed experiments, data interpretation, writing and editing of manuscript. VC: performed experiments, data acquisition, and interpretation, revision of manuscript. AL, RL: clinical sample collection, interpretation of clinical data and writing of manuscript. JA: study design, interpretation of clinical data, writing and editing of manuscript. FS: interpretation of data and critical revision of manuscript. AW: acquisition and FC data interpretation and writing of manuscript. KH: sample collection and preparation and revision of manuscript. LP: conception, contributed reagents, materials, analysis tools, and revision of manuscript. CO: conception, study design, experiments, data acquisition and interpretation, contributed reagents and materials, writing and editing of manuscript.

## Conflict of Interest Statement

The authors declare that the research was conducted in the absence of any commercial or financial relationships that could be construed as a potential conflict of interest.
