# Supplementary material for: Human Bronchial Epithelial Cells Induce CD141/CD123/DC-SIGN/FLT3 Monocytes That Promote Allogeneic Th17 Differentiation
Source: Front Immunol. 2017 Apr 25;8:447. doi: 10.3389/fimmu.2017.00447 (PMC5403901; doi:10.3389/fimmu.2017.00447)
Supplement: Supplementary file 1 [file Data_Sheet_1.DOC]

**Supplemental experimental procedures.**

**Culture of Primary human nasal epithelial cells (PNECs).**

PNEC were obtained by brushing the inferior surface of the middle turbinate of both nostrils using a cytological brush (Dent-o-care, London, UK) After brushing, brushes with cells were placed in a falcon tube containing 6 ml of PBS. Tubes containing brushes were vortexed for 5-10 seconds to remove cells from the brushes. The end of a 200μl pipet tip was cut with a sterile scissor and with the help of a pair of tweezers the tip was held into a Falcon tube containing PBS. Brushes were passed gently through the pipet tip 10-15 times, in order to remove remaining cells from the brush. The tubes containing PBS and nasal cells were centrifuged at 300g for 5 minutes. PBS was aspirated and cells were resuspended in 1 ml of Bronchial Epithelial Basal Medium (BEBM, Lonza, Switzerland) supplemented with Primocin (100μl/ml; InvigoGen, US) and Single Quots (SQ, Lonza, Switzerland). Single Quots contain BPE (bovine pituary extract), Insulin, hydrocortisone (HC), gentamycin and amphotericin (GA-1000), retinoic acid, transferrin, triiodthyronine, epinephrine and hEGF (human epidermal growth factor). Additional 2 ml of BEBM were added and placed on a pre-coated 12.5cm^2^ flask (Falcon, US). Prior to cell culture flasks were coated with a solution of collagen (Pure Col; Ceyllsystems, Germany) diluted 1:15 in sterile water. Cells were incubated at 37°C, 5% CO_2_. Twenty-four hours after isolation cells were washed with pre-warmed PBS prior to adding fresh BEBM. Medium was changed every 2-3 days following initial medium change until cells reached 80% of confluency. Prior to passaging, medium was aspirated and cells were washed once with PBS. One millilitre of trypsin/EDTA 0.25% was added to the flask and cells were incubated for 5 min at 37°C, 5% CO_2_. The flask was verified under the microscope, for complete detachment cells from the substrate. To inhibit further trypsin activity, 3ml of PBS and FCS (fetal calf serum, Amimed, BioConcept) 1:10 was added to the cells and suspension as transferred to a falcon tube, which was centrifuged at 300g for 5 minutes, the supernatant was aspirated and the cell pellet resuspended in 1 ml of BEBM. Cells were counted in Trypan blue in a Neubauer chamber and a minimum of 100’000 cells/well were seeded on 12 well inserts with 0.4 μm pore size (Nunc, Rochester, USA) and supplemented with 500ul of BEBM in the apical and 1ml of BEBM in the basal chamber. After 3-4 days, the cells reached 100% confluency. BEBM was removed and inserts were washed with 500 μl PBS. To the basal chamber 750 μl of Maitenance medium (750 μl PneumaCult-ALI, 10 μl Maitenance, 5 μl Hydrocortison, 2 μl Heparin, 1.5 μl Primorcin) was added, while the apical side of the cells was kept at the air-liquid interface. Air-liquid-cultures were washed with PBS once a week to remove mucus secreted at the apical side. Maintenance medium was changed every 2-3 days.
