# Supplementary figures and images for: Human Bronchial Epithelial Cells Induce CD141/CD123/DC-SIGN/FLT3 Monocytes That Promote Allogeneic Th17 Differentiation
Source: Front Immunol. 2017 Apr 25;8:447. doi: 10.3389/fimmu.2017.00447 (PMC5403901; doi:10.3389/fimmu.2017.00447)

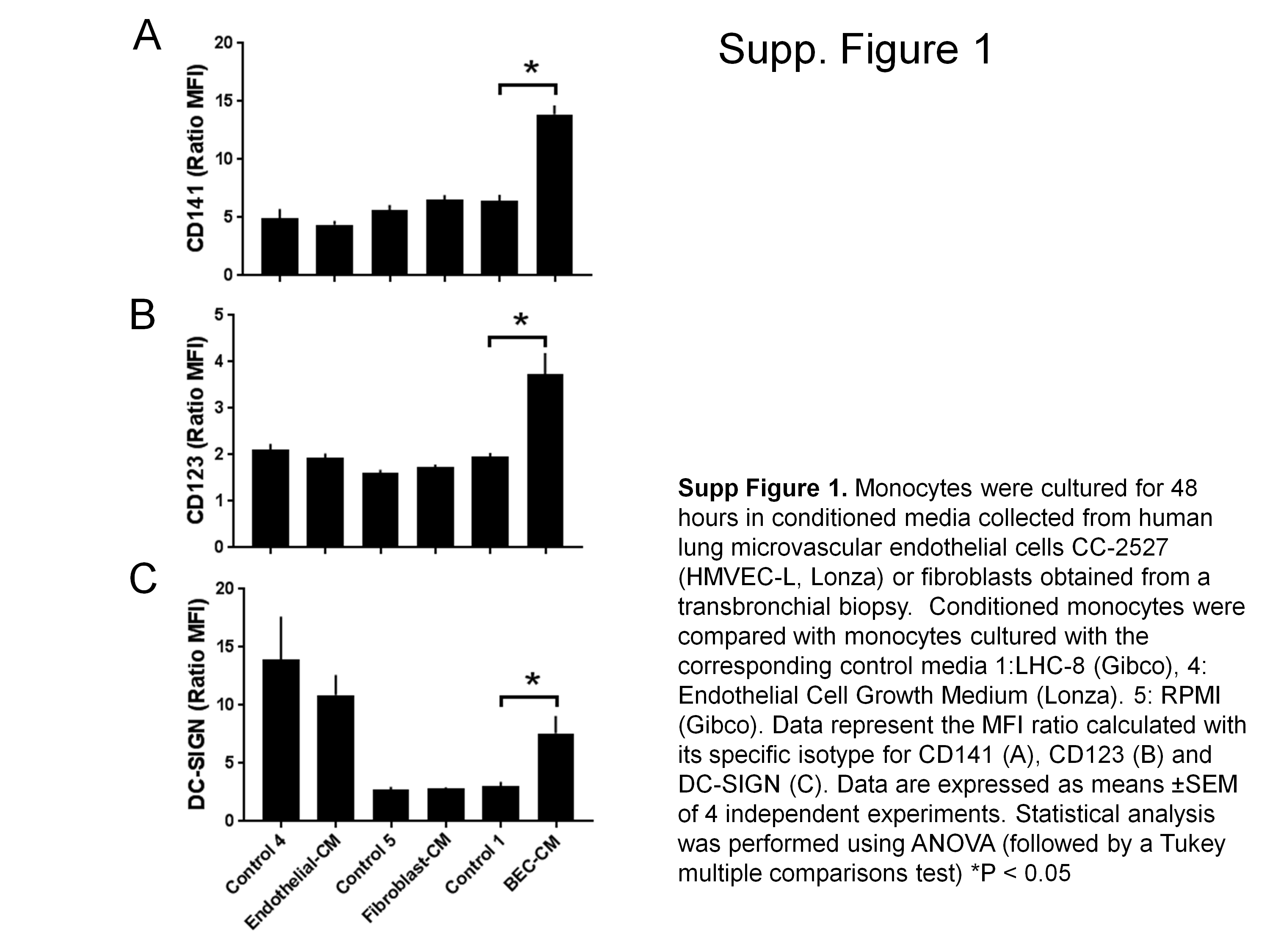

Supplement: Supplementary file 2 [file Image_1.TIF]

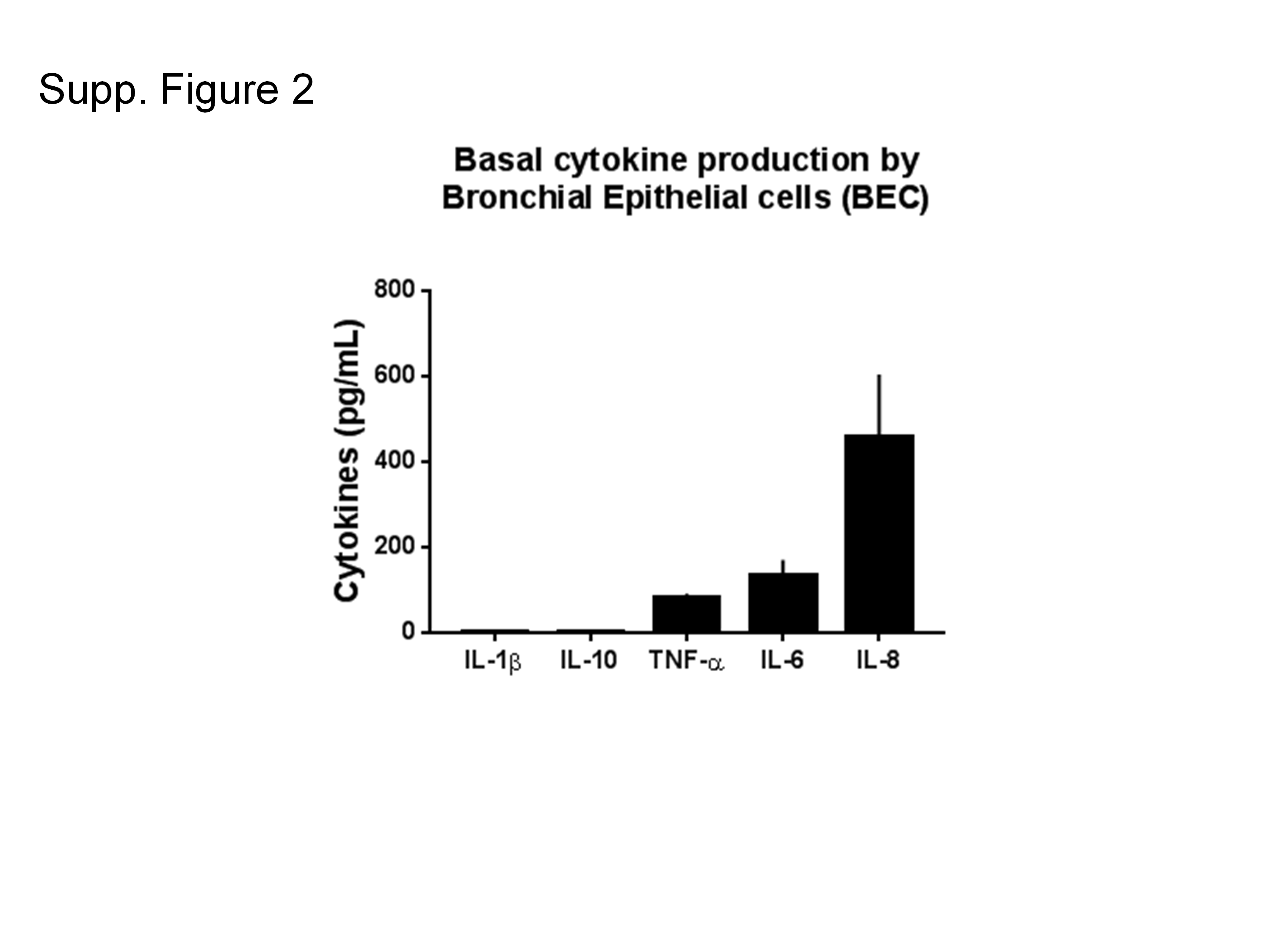

Supplement: Supplementary file 3 [file Image_2.TIF]

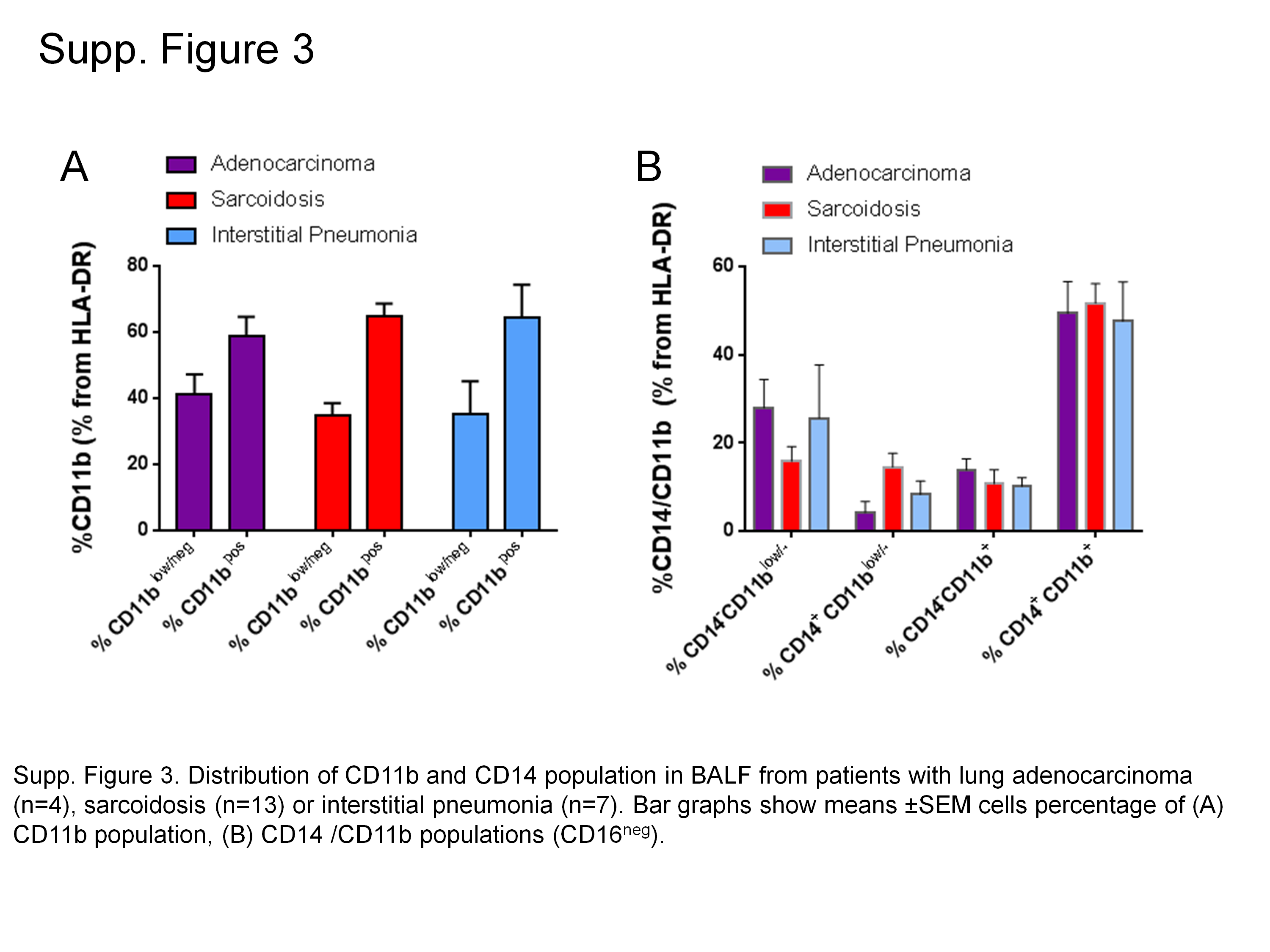

Supplement: Supplementary file 4 [file Image_3.TIF]

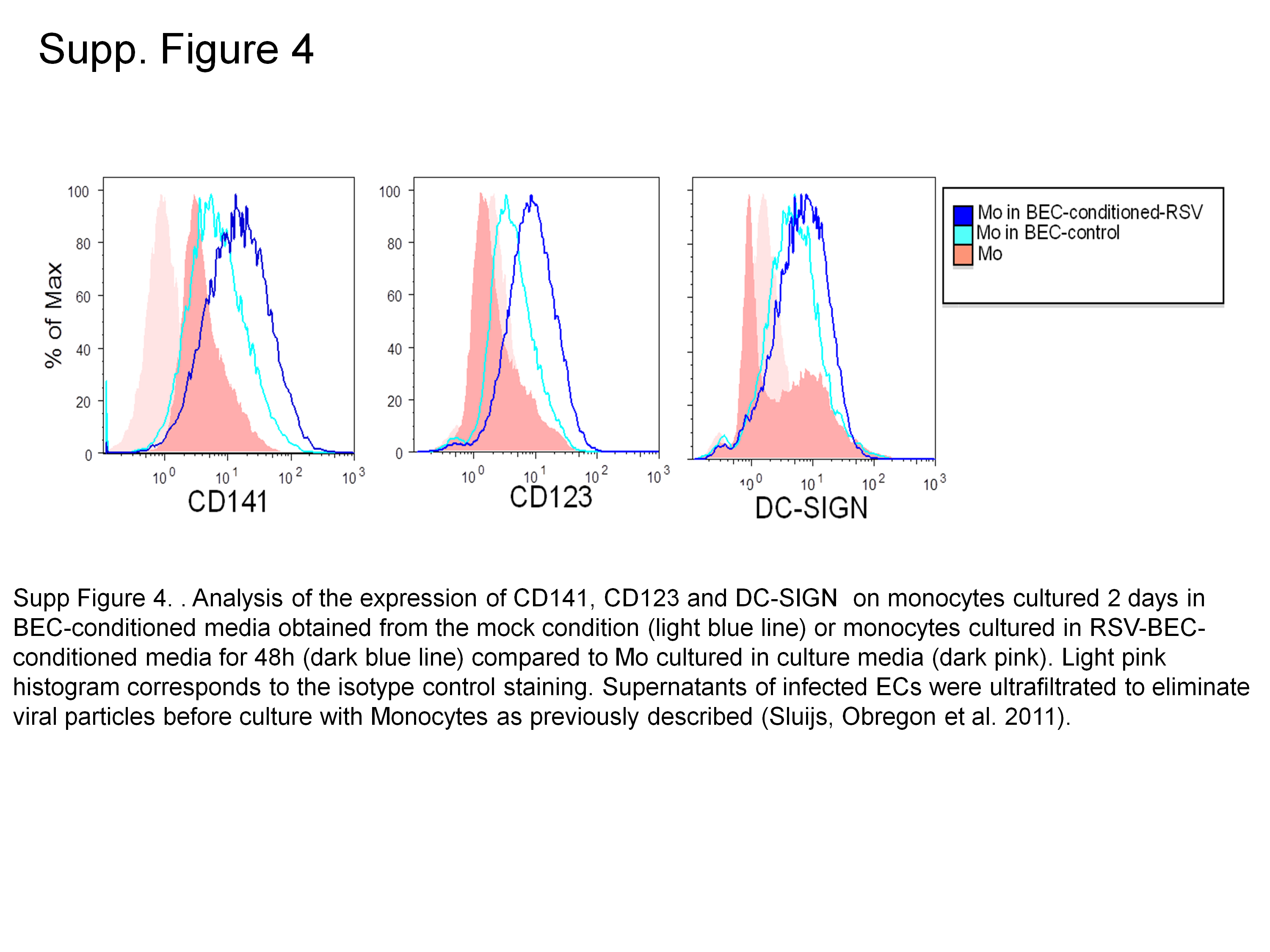

Supplement: Supplementary file 5 [file Image_4.TIF]
